# Preserving Life: How Retinoic Acid (RA) Enhances Cell Viability and Reduces Apoptosis in Cryopreserved Blastocyst Cells of Pudong Chickens

**DOI:** 10.3390/cells14070504

**Published:** 2025-03-28

**Authors:** Lingwei Sun, Fuqin Liu, Mengqian He, Jiehuan Xu, Caifeng Wu, Shushan Zhagn, Jun Gao, Jianjun Dai

**Affiliations:** 1 Shanghai Municipal Key Laboratory of Agri-Genetics and Breeding, Institute of Animal Science and Veterinary Medicine, Shanghai Academy of Agricultural Sciences, Shanghai 201106, China; sunlingwei1987@126.com (L.S.); lf21798@163.com (F.L.); he1037247863@163.com (M.H.); jiehuanxu810@163.com (J.X.); wucaifengwcf@163.com (C.W.); smalltreexj@126.com (S.Z.); 2 Key Laboratory of Livestock and Poultry Resources (Pig) Evaluation and Utilization, Ministry of Agriculture and Rural Affairs, Shanghai 201106, China

**Keywords:** retinoic acid (RA), blastoderm cells (BCs), cryopreservation, cell viability, cell adhesion, apoptosis, cell cycle, mitochondrial function

## Abstract

The preservation of chicken embryonic cells is essential for protecting avian genetic resources and enhancing breeding programs. This study investigates the effects of retinoic acid (RA) on the viability, functionality, and adhesion of thawed chicken blastoderm cells (BCs) following cryopreservation. After thawing and culturing the cells for 24 h, RA treatment resulted in significantly higher cell viability and adhesion rates compared to the control group, with the 2.0 μM RA group demonstrating the best outcomes. After 48 and 72 h of culture, similar trends were observed, with the 2.0 μM RA group consistently maintaining the highest cell viability and adhesion rates. Furthermore, immunofluorescence TUNEL assays revealed that RA significantly reduced both early and late apoptosis rates, particularly at a concentration of 2.0 μM, which exhibited a strong protective effect. Flow cytometry analysis indicated that RA treatment enhanced the mitochondrial membrane potential (MMP), reflecting improved cellular health. Analysis of the apoptosis-related genes BAX, BCL-2, and Caspase-3 revealed that moderate RA concentrations promoted the expression of anti-apoptotic factors while also upregulating pro-apoptotic factors, with the 2.0 μM RA group exhibiting the highest expression levels. Cell cycle analysis showed that RA significantly influenced the distribution of BCs across different phases, with the 4.0 μM RA group exhibiting the highest proportion of cells in the G1/G0 phase, suggesting an enhanced tolerance to cryopreservation stress. Conversely, the S phase cell population was notably reduced at higher RA concentrations, indicating potential inhibition of cell proliferation. These results suggest that RA not only significantly enhances the survival rates and mitochondrial function of BCs, but also regulates the cell cycle, providing better conditions for BC cryopreservation. Overall, the addition of RA represents a valuable strategy for optimizing cryopreservation techniques in chicken embryonic cells, with implications for avian genetic resource conservation and breeding strategies.

## 1. Introduction

According to the International Union for Conservation of Nature Red List, approximately 1.5% of bird species have gone extinct since 1500, while 13% of extant species are currently threatened [1]. Separately, the Food and Agriculture Organization reports that 32% of local chicken breeds are at risk of extinction due to agricultural genetic erosion [2]. The conservation of chicken genetic resources is essential to maintain economically valuable breeds for the sustainable production of eggs and meat. The biosecurity of chicken meat production is critical, especially given the vulnerability of chickens to infectious diseases such as avian influenza. The need to conserve and protect the genetic diversity of traditional chicken breeds has also been recognized. The genetic conservation of chickens is important not only for preserving the genetic diversity of extant non-commercial breeds but also for reintroducing these breeds to meet future demands [3]. However, most local chicken breeds are at risk of losing genetic diversity due to their small population sizes, making the conservation of chicken germplasm resources a pressing issue on both national and global scales. Small-scale efforts to cryopreserve rare breeds can be combined with commercial breeding to effectively conserve avian genetic resources.

Effective methods for conserving poultry biodiversity are urgently needed. Key approaches include the in vivo preservation and cryopreservation of semen, blastocyst cells (BCs), and primordial germ cells (PGCs) at ultra-low temperatures [4]. While cryopreservation saves time and costs, reduces pathogen risks, and preserves endangered species, traditional methods such as semen freezing are limited to adult male birds, resulting in low fertilization rates post-thawing. Furthermore, the cryopreservation of fertilized eggs is not feasible. Chicken BCs, classified as Stage X by the Eyal-Giladi and Kochav (EGK) system, are primordial germ cells which occur approximately 8–10 h post-ovulation and contain complete diploid genomes from both parents [5]. These BCs retain the ability to differentiate into male or female gametes, making them crucial for avian genetic resource conservation and serving as an alternative to haploid gametes. The long-term cryopreservation of poultry BCs in liquid nitrogen preserves parental genetic information, enabling organism restoration through chimeric technology. However, decreased cell viability post-freezing remains a challenge due to oxidative damage from excessive reactive oxygen species (ROS) during the freezing and thawing processes [6].

Antioxidants can mitigate this damage by neutralizing hydrogen peroxide (H_2_O_2_) and inhibiting elevated ROS levels, thereby protecting cellular integrity. Retinoic acid (RA), a group of natural or synthetic vitamin A analogs, plays a vital role in mammalian reproduction, embryogenesis, cell proliferation, differentiation, and apoptosis [7]. Previous studies have reported that RA maintains the redox balance of spermatozoa, enhancing antioxidant enzyme activities and improving tolerance to oxidative stress [8]. However, its effects on the freezing of chicken BCs have not been previously explored. Additionally, RA has been shown to promote the expression of germline-specific genes in BCs cultured in vitro, potentially increasing their germline transmission capacity [9].

This study aims to investigate the preservation of avian germplasm resources by focusing on chicken BCs. We hypothesize that the addition of different concentrations of RA to the cryoprotectant will enhance the cryopreservation outcomes of Pudong chicken BCs. To evaluate the effects of RA on BC cryopreservation, we will assess several key parameters after thawing, including cell viability, adhesion rates after 24 h of incubation, mitochondrial function, apoptosis levels, and cell cycle distribution. By elucidating the biochemical mechanisms underlying RA’s protective effects, this research seeks to provide valuable insights into cryopreservation technology for chicken BCs and to explore potential antioxidant applications in cell freezing.

## 2. Materials and Methods

### 2.1. Fertilized Eggs and Animal Care

Fertilized eggs from Pudong chickens were sourced from Shanghai Puhui Pudong Chicken Breeding Co. (Shanghai, China). The animal care and procedures in this study adhered to the guidelines set forth by the Animal Care and Use Committee of the Shanghai Academy of Agricultural Sciences.

### 2.2. Isolation and Culture of BCs

Chicken blastoderm cells (BCs) were individually collected from Stage X embryos, as previously outlined [10]. In total, 20 embryos were utilized for each experiment. The egg white was carefully removed from the yolk surface, and the yolk skin was covered with a filter paper ring. The yolk membrane was then cut around this ring with scissors and gently washed twice using calcium- and magnesium-free DPBS (Sigma-Aldrich, Taufkirchen, Germany). Following this, BCs were transferred to a clean solution of calcium- and magnesium-free DPBS to eliminate as much yolk as possible. The area pellucida was collected, gently pipetted, and centrifuged at 300× *g* for 5 min at 4 °C after cleaning. The supernatant was discarded, and the cells were resuspended at a density of 5 × 10^4^ cells/mL in 96-well flat-bottom cell culture plates. The cell culture was maintained in Dulbecco’s Modified Eagle Medium (DMEM; Life Technologies, Carlsbad, CA, USA) and supplemented with 10% fetal bovine serum (FBS; Gibco Invitrogen Co., Grand Island, NY, USA) and 1% penicillin/streptomycin (100 U/mL penicillin and 100 μg/mL streptomycin; Sigma Chemicals, Perth, Australia; cat. no. P0781). The cells were incubated at 37 °C in an atmosphere of 5% CO_2_ and 95% air. The culture medium was initially changed after 24 h and subsequently every 48 h. The cells were passaged every 2–3 days until they reached approximately 90% confluence.

### 2.3. Cryopreservation and Thawing of BCs

The cultured BCs were randomly divided into five treatment groups with five replicates per treatment group. Then, the BC suspension was dispensed into 1.2 mL cryotubes at a density of 8 × 10^5^ cells/mL and cryopreserved in cryoprotectant solution. The cryoprotectant solution contained 20% dimethyl sulfoxide (DMSO, Sigma Chemicals, Perth, Australia), 20% FBS, and 60% DMEM. Different concentrations of RA (Merck Millipore, Bedford, MA, USA; 0 μM, 0.5 μM, 1 μM, 2 μM, and 4 μM) were added to each of the five treatment groups. As soon as the samples were collected, they were immediately placed into a freezing vessel (Bicell, Nihon Freezer, Tokyo, Japan) and stored at −80 °C for 24 h before being transferred into liquid nitrogen.

Following a minimum storage period of four weeks in liquid nitrogen, the BC samples were subjected to thawing in a water bath maintained at 37 °C for one minute. Subsequently, the samples underwent centrifugation to eliminate the DMSO, after which they were resuspended in 300 µL of culture medium prior to their addition to the culturing plates. After an incubation period of 24 h, the cells were collected for further assays.

### 2.4. Post-Thaw Evaluation of BCs

#### 2.4.1. Cell Viability Measurement

The Cell Counting CCK-8 Kit (Beyotime, Shanghai, China) was used to determine the cell viability. The BCs (5000 cells per well) in each group were inoculated into 96-well plates and incubated for 24, 48, or 72 h under standard culture conditions (37 °C, 5% CO_2_). For the 48 and 72 h groups, 50% of the original culture medium was replaced with fresh pre-warmed medium containing different concentrations of RA at 24 h intervals to avoid nutrient depletion. At each time point (24/48/72 h), 10 μL of CCK-8 reagent was added to each well and incubated for 30 min at 37 °C. A microplate reader (BMG Labtech, Ortenberg, Germany) was used to measure the absorbance at 450 nm. Three independent replicates were performed for each time point.

#### 2.4.2. Adherent Cell Analysis

Following the thawing process, the cells were subjected to incubation in the cell culture medium for a duration of 24 h. The adherent cells in each well were subsequently examined and assessed utilizing an inverted fluorescence microscope. For the 48 and 72 h assessments, 50% of the original culture medium was replaced with fresh pre-warmed medium containing different concentrations of RA at 24 h. To determine the percentage of adherent cells at each time point (24, 48, and 72 h), the following equation was utilized: % Adherent Cells = (Number of adherent cells/Total cell count) × 100%.

#### 2.4.3. Flow Cytometric Analysis of Mitochondrial Membrane Potential (MMP)

The MMP loss of BCs was assessed using a JC-1 kit (Keygen, Nanjing, China). Briefly, the cells were incubated with JC-1 staining solution for 20 min at 37 °C in the dark, followed by two washes with incubation buffer. Fluorescence images of JC-1 monomers and aggregates were captured using a FACSCalibur flow cytometer (BD Biosciences, San Jose, CA, USA) with an excitation wavelength of 488 nm and emission wavelengths of 529 nm for monomers and 590 nm for aggregates. MMP was characterized by calculating the ratio of aggregates (red fluorescence) to monomers (green fluorescence).

#### 2.4.4. Apoptosis Analysis

Apoptosis was evaluated through flow cytometric analysis employing Annexin V–fluorescein isothiocyanate (FITC) apoptosis detection kits (Solarbio, Beijing, China), following the manufacturer’s protocol. Initially, the cells were washed with PBS and resuspended in coupling buffer. Subsequently, the cells were incubated with 250 ng/mL of Annexin V–FITC for 15 min at a temperature of 4 °C and shielded from light exposure. Following this, the cells underwent another wash with PBS and were resuspended in 190 µL of coupling buffer. Then, the cells were treated with 10 µL of propidium iodide (PI) for a duration of 5 min in darkness. The staining of cardiomyocytes was analyzed via flow cytometry. The data were processed using a flow cytometer, which enabled the identification of apoptotic cells, including early apoptotic cells (characterized by Annexin V positivity and PI negativity) and late apoptotic cells (identified as being both Annexin V- and PI-positive). This methodology utilizing flow cytometry facilitated a quantitative assessment of apoptosis, thereby providing a definitive differentiation among the various stages of apoptosis across a substantial cell population.

In order to further elucidate the mechanisms underlying apoptosis, particularly the late-stage apoptotic alterations, the Terminal Deoxynucleotidyl Transferase-Mediated dUTP End Labeling (TUNEL) assay was implemented. The cells were cultivated following a previously established protocol. They were subsequently fixed in 4% paraformaldehyde for 30 min, followed by rinsing with PBS and a brief incubation in ice-cold 0.1% Triton X-100 for 2 min. Fresh TUNEL detection buffer was then introduced, and the cells were incubated in a dark environment at 37 °C for a duration of 60 min. After this incubation, the cells underwent an additional wash with PBS. Ultimately, the observations were conducted using an Olympus FV3000 confocal microscope, with data analysis carried out employing the ImageJ software (version 1.30). The excitation wavelength for Cy3 was set at 550 nm, while the emission wavelength was recorded at 570 nm, as specified by the One Step TUNEL Apoptosis Assay Kit (Beyotime, Beijing, China). Cells that exhibited red fluorescence were identified as being TUNEL-positive. The TUNEL assay is specifically designed to detect DNA fragmentation, a significant marker of late-stage apoptosis, thereby enhancing the insights gained from flow cytometric analysis and providing a more holistic view of the apoptotic phenomena occurring within the cells.

#### 2.4.5. Expression Analysis of Apoptosis-Related Genes

To assess the expression of apoptosis-related genes in BCs, total RNA was extracted using TRIzol reagent (Invitrogen, Carlsbad, CA, USA) according to the manufacturer’s instructions. Cells were harvested, lysed in TRIzol, and chloroform was added to separate the RNA from the proteins and DNA. The RNA was precipitated with isopropanol, washed with 75% ethanol, air-dried, and dissolved in RNase-free water. RNA concentration and purity were measured using a NanoDrop spectrophotometer (Thermo Fisher Scientific, Waltham, MA, USA). Complementary DNA (cDNA) was synthesized from 1 µg of total RNA using the PrimeScript™ RT Reagent Kit (Takara, Dalian, China). The reverse transcription was conducted at 37 °C for 15 min, followed by inactivation at 85 °C for 5 s. Quantitative PCR (qPCR) was performed to quantify the expression levels of anti-apoptotic protein B-cell lymphoma 2 (Bcl-2), Bcl-2 Associated X protein (BAX), and Caspase-3 using gene-specific primers [11], with β-actin as an internal control. Each qPCR reaction contained 10 µL of SYBR Green PCR Master Mix (Applied Biosystems, Foster City, CA, USA), 0.5 µM of each primer, and 2 µL of cDNA template in a total volume of 20 µL. The amplification protocol included an initial denaturation at 95 °C for 10 min, followed by 40 cycles of denaturation at 95 °C for 15 sec and annealing/extension at 60 °C for 1 min. The specific annealing temperatures for each gene are listed in Supplementary Table S1. The relative expression levels were calculated using the 2^−ΔΔCt^ method, and all experiments were performed in triplicate.

#### 2.4.6. Flow Cytometric Analysis of Cell Cycle Distribution

Briefly, the BCs underwent a washing process using ice-cold PBS followed by fixation in 70% ethanol overnight at −20 °C. Subsequently, the cells were rinsed with PBS for 10 min and treated with RNase A at a concentration of 1 mg/mL for 30 min at 37 °C. After this incubation, the cells were stained with PI in combination with RNase. The analysis of the cell cycle distribution was performed utilizing Cell Quest software (latest v. 5.2.1, BD Biosciences, Franklin Lakes, NJ, USA).

### 2.5. Statistical Analysis

The data were evaluated employing IBM SPSS Statistics version 20.0 (SPSS Inc., Chicago, IL, USA). A one-way analysis of variance (ANOVA) was conducted to determine the presence of significant differences among the various groups, which was subsequently accompanied by post hoc analysis through the application of the LSD multiple comparisons test. All data were presented as the mean ± S.E.M., with a significance threshold established at *p* < 0.05. Histograms were generated using GraphPad Prism 6 (GraphPad Software Inc., San Diego, CA, USA).

## 3. Results

### 3.1. Effects of RA Treatment on the Viability and Adhesion of Thawed Chicken BCs

**Figure 1:**
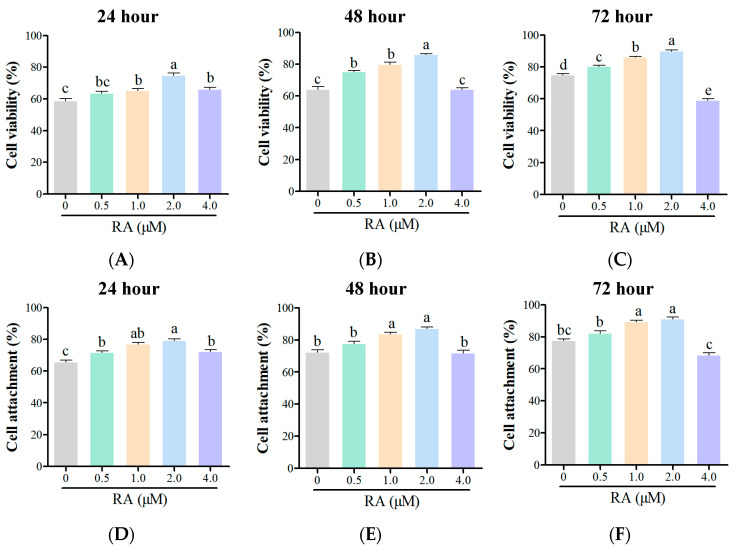
Effects of retinoic acid (RA) treatment on the viability and adhesion of thawed chicken blastoderm cells (BCs). Cell viability rates ((**A**): 24 h, (**B**): 48 h, (**C**): 72 h) and adhesion rates ((**D**): 24 h, (**E**): 48 h, (**F**): 72 h) were analyzed after thawing and culturing across different RA concentrations. Data are presented as the mean ± S.E.M. (*n* = 5 per group). Different letters in the bar graph indicate significant differences (*p* < 0.05).

After thawing the chicken embryonic cells and culturing them for 24 h, the cell viability in the different treatment groups was analyzed (Figure 1A). The findings indicated that the viability of cells in the control group was markedly reduced compared to the treatment groups that included RA. Specifically, the group with 2.0 μM RA exhibited the highest cell viability. The group with 1.0 μM RA also showed a high viability, although it was lower than the 2.0 μM group. In contrast, the group with 4.0 μM RA demonstrated a decrease in cell viability, indicating a potential inhibitory effect at this higher concentration. After thawing and culturing the cells for 48 h, similar trends were observed (Figure 1B). The control group without RA treatment continued to show a significantly lower viability compared to the RA treatment groups. The 2.0 μM RA group maintained the highest cell viability, while the 1.0 μM RA group also exhibited good viability but remained lower than the 2.0 μM group. The 4.0 μM RA group again showed decreased viability. After 72 h of culture, the viability results continued to reflect the trends observed at the earlier time points, with the 2.0 μM RA group consistently demonstrating the highest viability (Figure 1C).

Regarding the cell adhesion rates, after 24 h, all RA treatment groups exhibited significantly higher adhesion rates compared to the control group (Figure 1D). The 2.0 μM RA group again showed the best adhesion ability, while the 1.0 μM RA group also had a high adhesion rate, although lower than that of the 2.0 μM group. The 0.5 μM and 4.0 μM RA groups had significantly lower adhesion rates compared to the 2.0 μM RA group. After 48 h, this trend persisted, with all RA treatment groups again showing higher adhesion rates than the control group (Figure 1E). The 2.0 μM RA group maintained its superior adhesion capabilities, while the higher concentration of 4.0 μM indicated a negative impact on cell adhesion, consistent with the previous observations. This pattern continued after 72 h (Figure 1F), reinforcing the conclusion that appropriate concentrations of RA can enhance both cell viability and adhesion, while excessively high concentrations may inhibit these processes.

### 3.2. Effects of RA Treatment on the Mitochondrial Function of Thawed Chicken BCs

**Figure 2:**
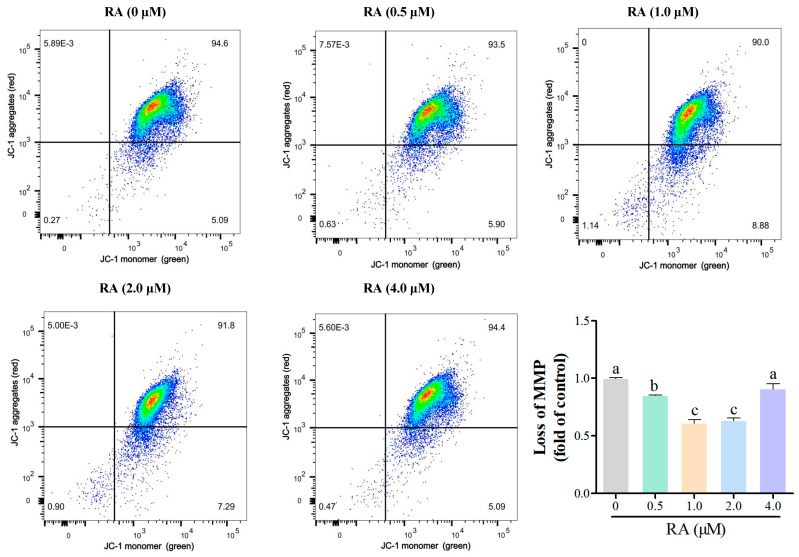
The effects of retinoic acid (RA) treatment on the loss of mitochondrial membrane potential (MMP) of thawed chicken blastoderm cells (BCs) were evaluated. Flow cytometry analysis was conducted to assess the loss of MMP after 24 h of culture. The histogram displays the specific proportion of loss of MMP in each group. Data are presented as the mean ± S.E.M. (*n* = 5 per group). Different letters in the bar graph indicate significant differences (*p* < 0.05).

Flow cytometry was further used to analyze the loss of mitochondrial membrane potential (MMP) of chicken embryonic cells after thawing and culturing for 24 h, with the results shown in Figure 2. Except for the 4.0 μM RA group, the loss of MMP in the cells from other RA treatment groups was significantly lower than that of the control group, indicating that an appropriate concentration of RA can effectively enhance the mitochondrial membrane potential of cells, reflecting their health status. Among the groups with different RA concentrations, the 1.0 μM and 2.0 μM RA groups exhibited the lowest loss of MMP, and the difference between these two groups was not significant, suggesting that the maintenance of mitochondrial membrane potential may be inhibited at these two concentrations. These results indicate that the concentration of RA significantly affects the mitochondrial function of chicken embryonic cells.

### 3.3. Effects of RA Treatment on the Apoptosis of Thawed Chicken BCs

**Figure 3:**
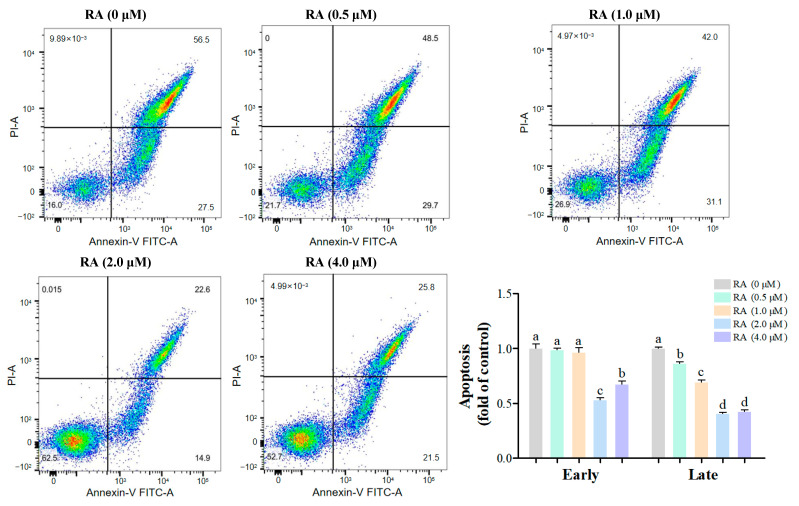
Early and late apoptosis rates in thawed chicken blastoderm cells (BCs) after RA treatment. Flow cytometric analysis was performed to detect early and late apoptosis following thawing and culturing for 24 h. Data are presented as the mean ± S.E.M. (*n* = 5 per group). Different letters in the bar graph indicate significant differences (*p* < 0.05).

Early and late apoptosis play different roles in the quality of cell cryopreservation. Early apoptosis may affect cell survival and function, but its effects are relatively mild, whereas late apoptosis usually leads to irreversible cell death, significantly reducing the quality of cryopreservation. In this experiment, the early and late apoptosis of chicken embryonic cells after thawing and culturing for 24 h was detected and analyzed by flow cytometry (Figure 3). In the detection of early apoptosis, we found that there was no significant difference between the 0.5 μM and 1.0 μM RA groups and the control group, indicating that RA had a minor effect on early apoptosis at these two concentrations. However, the early apoptosis rates in the 2.0 μM and 4.0 μM RA groups were significantly lower than that of the control group, with the 2.0 μM RA group showing the lowest early apoptosis rate, indicating a strong protective effect on cells. Further analysis of late apoptosis results showed that the late apoptosis rates in all RA treatment groups were significantly lower than that of the control group, indicating that the addition of RA effectively reduced late apoptosis in cells. Specifically, the late apoptosis rates in the 2.0 μM and 4.0 μM RA groups were significantly lower than those in the 0.5 μM and 1.0 μM RA groups, while the difference between these two groups was not significant, suggesting that at higher concentrations, the protective effect of RA on cells tends to saturate.

**Figure 4:**
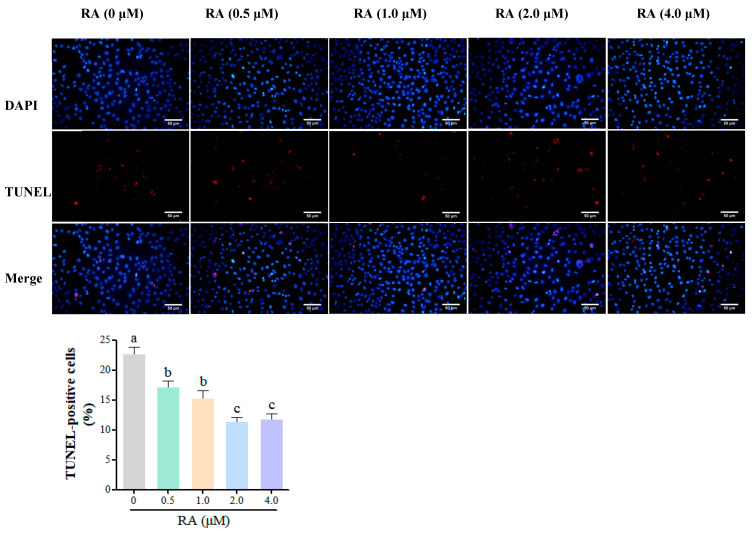
TUNEL detection of apoptosis in thawed chicken blastoderm cells (BCs) treated with different concentrations of retinoic acid (RA). The percentage of apoptosis was assessed and compared to the control group. Data are presented as the mean ±S.E.M. (*n* = 5 per group). Different letters in the bar graph indicate significant differences (*p* < 0.05).

This experiment also conducted immunofluorescence TUNEL detection to assess the effects of different concentrations of RA treatment on cell apoptosis. The results are shown in Figure 4, where the percentage of apoptosis in all RA treatment groups was significantly lower than that of the control group (*p* < 0.01), indicating that RA treatment could effectively reduce cell apoptosis. Furthermore, as the concentration of RA increased, the percentage of cell apoptosis showed a gradually decreasing trend, suggesting a negative correlation between RA concentration and cell apoptosis. Particularly in the 2.0 μM and 4.0 μM RA groups, the percentage of cell apoptosis reached the lowest level, further validating the effectiveness of higher concentrations of RA in inhibiting cell apoptosis. These results indicate that RA can not only regulate the cell cycle but also significantly improve cell survival rates, potentially providing better conditions for cell cryopreservation.

### 3.4. Effects of RA Treatment on the Expression of Apoptosis-Related Genes in Thawed Chicken BCs

**Figure 5:**
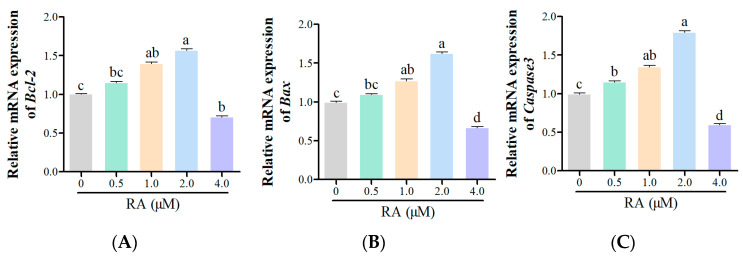
Expression of apoptosis-related genes of thawed chicken blastoderm cells (BCs) under different concentrations of retinoic acid (RA). (**A**): B-cell lymphoma 2 (BCL-2); (**B**): Bcl-2 Associated X protein (BAX); (**C**): Caspase3. Data are presented as the mean ± S.E.M. (*n* = 5 per group). Different letters in the bar graph indicate significant differences (*p* < 0.05).

The effects of RA treatment on the expression of apoptosis-related genes BAX, BCL-2, and Caspase-3 in thawed chicken BCs were evaluated (Figure 5). Following treatment with 0.5 μM RA, a slight increase in the expression of BAX and BCL-2 was observed, along with a modest rise in Caspase-3 expression, indicating the minor enhancing effect of low RA concentration on these genes. At 1.0 μM RA, the expression levels of BAX and BCL-2 significantly increased, while Caspase-3 expression also demonstrated a notable rise. This suggests that moderate concentrations of RA promote the expression of anti-apoptotic factors while simultaneously upregulating pro-apoptotic factors. The 2.0 μM RA treatment group exhibited the highest expression levels for BAX and BCL-2, alongside a significant increase in Caspase-3 expression. These findings indicate that this concentration of RA markedly enhances the expression of pro-apoptotic genes while maintaining a balance with anti-apoptotic signals. Conversely, in the 4.0 μM RA group, the expression levels of all three genes decreased significantly.

### 3.5. Effects of RA Treatment on the Cell Cycle Distribution of Thawed Chicken BCs

**Figure 6:**
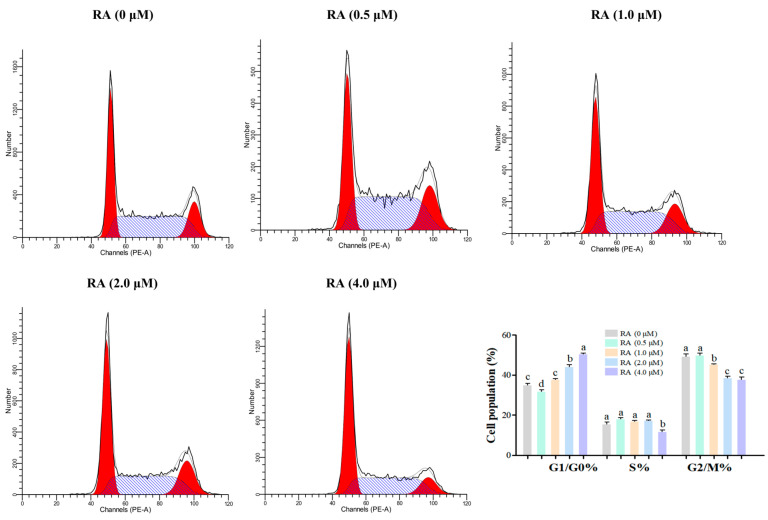
Cell cycle distribution of thawed chicken blastoderm cells (BCs) under different concentrations of retinoic acid (RA). The prominent red peak on the left represents cells in the G1 phase, while the smaller secondary red peak on the right corresponds to cells in the G2 phase. The diagonal blue hatched area indicates cells in the S phase. The histogram shows the distribution of cells in the G1/G0, S, and G2/M phases of the cell cycle under different concentrations of RA. Data are presented as the mean ± S.E.M. (*n* = 5 per group). Different letters in the bar graph indicate significant differences (*p* < 0.05).

There is a significant correlation between the cell cycle and the quality of cell cryopreservation. The stage of the cell cycle has a crucial impact on the sensitivity, survival rate, and functional recovery ability of cells during cryopreservation. The experimental results of this study show (Figure 6) that there are significant differences in the distribution of cells in various stages of the cell cycle under different concentrations of RA treatment. Except for the 0.5 μM RA group, the proportion of cells in the G1/G0 phase in the other RA treatment groups was significantly higher than that of the control group, with the 4.0 μM RA group having the highest proportion of G1/G0 phase cells, indicating that higher concentrations of RA can effectively promote cell arrest in the G1/G0 phase, which helps to improve the cells’ tolerance to cryopreservation. In a further analysis of the proportion of S phase cells, only the 4.0 μM RA group had a significantly lower proportion of S phase cells compared to other treatment groups, while there was no significant difference between the 0.5 μM and 2.0 μM RA groups and the control group. This result suggests that higher concentrations of RA may inhibit cell proliferation in the S phase, affecting the cells’ proliferative capacity. Finally, when analyzing the proportion of G2/M phase cells, the results showed that, except for the 0.5 μM RA group, the proportion of cells in the G2/M phase in other RA treatment groups was significantly lower than that of the control group, with the 2.0 μM and 4.0 μM RA groups having the lowest proportions. This indicates that the addition of RA may promote the accumulation of cells in the G2/M phase, which is beneficial for cell division and proliferation.

## 4. Discussion

Research has shown that cells may experience various forms of damage during cryopreservation, including membrane damage, ice crystal formation, and oxidative stress, all of which negatively impact their subsequent biological functions [12]. To improve the quality of cells post-cryopreservation, exploring effective protective agents has become a focal point of research. RA, an important bioactive molecule, has been shown to play a critical role in promoting cell survival and functionality [13]. Previous studies have shown that appropriate concentrations of RA can promote cell survival by modulating intracellular signaling pathways, including those involved in apoptosis and cell cycle regulation [14].

To improve the quality of cells post-cryopreservation, exploring effective protective agents has become a focal point of research. RA, an important bioactive molecule, has been shown to play a critical role in promoting cell survival and functionality [15]. The results of this study demonstrate that the addition of RA significantly enhances the viability and adhesion of thawed chicken embryonic cells, with 2.0 μM RA consistently exhibiting optimal effects across all time points (24–72 h). This finding aligns with previous studies indicating that appropriate concentrations of RA can promote neuronal cell survival by modulating intracellular signaling pathways [16]. The sustained improvement in cell viability during prolonged culture (72 h) can be attributed to RA’s ability to enhance survival rates, as demonstrated in fetal alveolar epithelial cells under extended culture conditions [16]. This mechanism is central to RA’s protective effects, as enhanced mitochondrial function directly supports cellular energy production—particularly critical during post-thaw recovery. Mitochondrial integrity ensures adequate ATP synthesis to meet the heightened metabolic demands of surviving cells, while also mitigating cryopreservation-induced oxidative stress. These dual roles explain why the 2.0 μM RA group maintained superior viability throughout the 72 h culture period, whereas the 4.0 μM group’s progressive viability loss likely reflected mitochondrial dysfunction under excessive RA exposure.

The role of RA in promoting cell adhesion is particularly important in the context of cell culture and tissue engineering, where effective cell attachment is crucial for maintaining cellular function and promoting tissue regeneration [17]. Additionally, RA’s influence on integrin expression, which plays a vital role in cell–matrix interactions, further supports its function in enhancing adhesion [18]. In terms of cell adhesion, all RA treatment groups exhibited significantly higher adhesion rates compared to the control group, with the 2.0 μM RA group showing the best adhesion ability. This result is consistent with the known effects of RA on promoting cell adhesion and migration, as demonstrated by [19]. It has been shown that RA enhances cell adhesion by upregulating the expression of extracellular matrix components, such as fibronectin and laminin, which are critical for cell attachment and stability on the substrate [20,21].

Furthermore, RA may enhance the cytoskeletal reorganization necessary for stable cell attachment, facilitating the formation of focal adhesions that anchor cells to the extracellular matrix [22]. This cytoskeletal remodeling is crucial for maintaining cell shape and motility, and studies have shown that RA can influence the dynamics of actin filaments and microtubules, thereby promoting effective cell adhesion and migration [23]. However, the adhesion rates in the 0.5 μM and 4.0 μM RA groups were significantly lower than those in the 2.0 μM group, suggesting a diminished promoting effect of RA on cell adhesion at these two concentrations. This may be attributed to changes in cellular sensitivity to RA, where lower concentrations may not provide sufficient signaling for optimal adhesion, while higher concentrations may lead to cellular stress or toxicity, impairing adhesion capabilities [24]. A previous study has indicated that excessive RA can induce cytotoxic effects, potentially disrupting normal cellular functions and compromising adhesion [25].

Moreover, the protective effects of RA extend beyond cell viability; it has been implicated in enhancing mitochondrial function, which is essential for energy production and overall cellular health [26]. This is particularly relevant in the context of cryopreservation, where maintaining mitochondrial integrity can significantly influence cell recovery and functionality post-thaw. Flow cytometry analysis revealed that RA treatment significantly decreased the MMP loss in cells, reflecting their health status. Except for the 4.0 μM RA group, the loss of MMP in other RA treatment groups was significantly lower than that of the control group, indicating that an appropriate concentration of RA could effectively enhance mitochondrial function [27]. A decrease in mitochondrial membrane potential loss is typically associated with improved cellular energy metabolism, suggesting that cells treated with RA can more effectively synthesize ATP [28,29]. However, the 1.0 μM and 4.0 μM RA groups exhibited the greatest loss of MMP, which may have indicated an inhibitory effect on mitochondrial function at these concentrations. This finding corroborates previous studies that suggest that excessive RA may lead to mitochondrial dysfunction, thereby affecting cellular energy metabolism [30,31].

Early apoptosis is considered an adaptive response of cells to environmental stress [32]. During cryopreservation, cells face various stressors, such as oxidative stress and membrane damage, which can trigger early apoptosis and reduce cell viability. Our study found that RA significantly decreased the early apoptosis rate of BCs, especially in the 2.0 μM RA group, suggesting that RA enhances the stress adaptation capacity of BCs and mitigates damage during freezing. This protective mechanism could be associated with the regulation of apoptosis-related proteins by RA, which includes the upregulation of the Bcl-2 and the suppression of pro-apoptotic proteins, ultimately leading to a reduction in early apoptotic events [33]. Additionally, RA may lower intracellular ROS production by modulating mitochondrial function [34]. Our results also demonstrated that RA significantly reduced the late apoptosis rate of BCs, particularly in the 2.0 μM and 4.0 μM treatment groups. This indicates that RA not only alleviates early apoptosis but also inhibits the progression of late apoptosis, improving the survival quality of BCs. RA appears to inhibit the activation of Caspases, thereby slowing the transition to late apoptosis [35]. A study has shown that RA can downregulate the expression of Caspase-3 and Caspase-9, further contributing to the reduction in apoptosis occurrence [28]. Using immunofluorescence TUNEL detection, we also found that the percentage of apoptosis in all RA treatment groups was significantly lower than in the control group, with a negative correlation between RA concentration and cell apoptosis. In particular, the 2.0 μM and 4.0 μM RA groups exhibited the lowest apoptosis rates, supporting the effectiveness of higher RA concentrations in inhibiting apoptosis. The protective effects of RA against apoptosis may stem from its modulation of key apoptotic pathways, including the regulation of Bcl-2 and the Caspase family [31,34]. RA upregulates Bcl-2 to maintain mitochondrial integrity, thereby reducing the likelihood of apoptosis [36].

The dual role of RA in regulating apoptosis-related genes (BAX, BCL-2, and Caspase-3) in thawed chicken BCs aligns with its concentration-dependent effects observed in other models. At lower concentrations (0.5–2.0 μM), RA enhanced cell survival by upregulating BCL-2 and suppressing BAX and Caspase-3, consistent with its anti-apoptotic effects in neuronal and hepatic cells [37]. This protective mechanism may have involved the RA-mediated stabilization of mitochondrial function. In contrast, 4.0 μM RA induced a pro-apoptotic shift, characterized by BCL-2 suppression and Caspase-3 activation, likely driven by oxidative stress or mitochondrial dysfunction. The paradoxical downregulation of BAX at 4.0 μM RA may have reflected compensatory feedback mechanisms, as seen in RA-treated HTLV-1-infected T-cells [38]. These findings highlight RA’s therapeutic potential for improving post-thaw cell survival at moderate concentrations, while underscoring the risks of high-dose toxicity.

The different stages of the cell cycle play a crucial role in determining cell survival and functional recovery [39]. Our results indicate that RA significantly affects the distribution of BCs across different stages of the cell cycle, particularly in the 4.0 μM RA group, which exhibited the highest proportion of cells in the G1/G0 phase. This phenomenon may be closely related to RA’s regulatory effects on cell growth and metabolism. Cells in the G1/G0 phase are typically in a quiescent state, demonstrating greater adaptability to environmental changes, which allows them to better cope with oxidative stress and cell membrane damage during cryopreservation, thereby enhancing their survival rate [40]. This mechanism may play a crucial role in reducing the cellular damage that occurs during the freezing process. Conversely, the significant reduction in S phase cells in the 4.0 μM RA group suggests that higher concentrations of RA may inhibit cell proliferation and affect their division capacity [41]. During the S phase, cells are in a critical stage of DNA replication and are particularly sensitive to external stressors; thus, the inhibitory effect of RA may expose cells to a higher risk of DNA damage after cryopreservation. Additionally, RA treatment (except 0.5 μM) reduced the proportion of G2/M phase cells compared to the control group, indicating that RA does not promote G2/M accumulation but instead restricts cell cycle progression. In summary, the concentration-dependent effects of RA on different stages of the cell cycle significantly impact the survival and functional recovery of BCs. An appropriate concentration of RA can effectively enhance cell survival, while excessively high concentrations may inhibit cell proliferation and affect functional recovery.

## 5. Conclusions

In summary, the addition of RA significantly enhances the viability, functionality, and adhesion of thawed chicken BCs, establishing it as a valuable agent for improving cryopreservation outcomes. Our results demonstrated that RA effectively promotes mitochondrial function and reduces apoptosis, highlighting its critical role in avian embryonic cell preservation. Notably, the optimal concentration of 2.0 μM RA exhibited the most pronounced protective effects, leading to enhanced cellular viability and adhesion compared to other concentrations. Furthermore, after 48 and 72 h of culture, the 2.0 μM RA group consistently maintained the highest cell viability and adhesion rates, reinforcing its effectiveness. This concentration was essential for maximizing cell viability and adhesion, emphasizing the importance of careful concentration selection in cryopreservation protocols. The preservation of chicken embryonic cells is crucial for safeguarding avian genetic resources and supporting breeding programs. Additionally, the analysis of the apoptosis-related genes BAX, BCL-2, and Caspase3 revealed that moderate RA concentrations promoted the expression of anti-apoptotic factors while upregulating pro-apoptotic factors, with the 2.0 μM RA group showing the highest expression levels. Future research should aim to elucidate the molecular mechanisms underlying RA’s protective effects and explore its applications in other avian species to optimize cryopreservation techniques further.

## Data Availability

Data are contained within the article.

## Supplementary Materials

The following supporting information can be downloaded at: https://www.mdpi.com/article/10.3390/cells14070504/s1. Table S1. Gene Primer Sequences.
