# Supplementary material for: Preserving Life: How Retinoic Acid (RA) Enhances Cell Viability and Reduces Apoptosis in Cryopreserved Blastocyst Cells of Pudong Chickens
Source: Cells. 2025 Mar 28;14(7):504. doi: 10.3390/cells14070504 (PMC11988042; doi:10.3390/cells14070504)
Supplement: Supplementary file 1 [file cells-14-00504-s001.zip › cells-3474315-supplementary.pdf]

Supplemental Table

Table S1. Gene Primer Sequences

Target genes	Forward primer (5'-3')	Length (bp)	Annealingtemperature (°C)
β-actin	F: AGGAGAAGCTGTGCTACGTC<br>R: TACCACAGGACTCCATACCCAA	183	60
Bax	F: GTGATGGCATGGGACATAGCTC<br>R: TGGCGTAGACCTTGCGGATAA	91	60
Bcl-2	F: ATCGTCGCCTTCTTCGAGTT<br>R: GTAGCACCTCCAACAAAAGA	151	58
Caspase3	F: AAGCGAAGCAGTTTTGTTTGTG<br>R: GCTAGACTTCTGCACTTGTCACCTC	128	60

[1] BAX: Bcl-2 Associated X protein; BCL-2: B-cell lymphoma 2.
